# Functional Analysis of β-Carotene Oxygenase 2 (*BCO2*) Gene in Yesso Scallop (*Patinopecten yessoensis*)

**DOI:** 10.3390/ijms25073947

**Published:** 2024-04-02

**Authors:** Shiqi Liu, Shuyue Wang, Liang Zhao, Tingting Li, Yihan Zhang, Huizhen Wang, Zhenmin Bao, Xiaoli Hu

**Affiliations:** 1 MOE Key Laboratory of Molecular Genetics and Breeding, College of Marine Life Sciences, Ocean University of China, Qingdao 266003, China; shiqiliu0110@163.com (S.L.); lchxs465@outlook.com (S.W.); z13905330705@163.com (Y.Z.);; 2 Laboratory for Marine Fisheries Science and Food Production Processes, Qingdao National Laboratory for Marine Science and Technology, Qingdao 266237, China; 3 Laboratory of Tropical Marine Germplasm Resources and Breeding Engineering, Sanya Oceanographic Institution, Ocean University of China, Sanya 572000, China

**Keywords:** Yesso scallop (*Patinopecten yessoensis*), *BCO2*, carotenoid, RNA interference, meat quality parameter

## Abstract

Carotenoids are essential nutrients for humans and animals, and carotenoid coloration represents an important meat quality parameter for many farmed animals. Increasingly, studies have demonstrated that vertebrate carotenoid cleavage oxygenases (*CCO*s) are essential enzymes in carotenoid metabolism and are therefore potential candidate genes for improving carotenoid deposition. However, our understanding of carotenoid bioavailability and *CCO*s functions in invertebrates, particularly marine species, is currently quite limited. We previously identified that a *CCO* homolog, *PyBCO-like 1*, was the causal gene for carotenoid coloration in the ‘Haida golden scallop’, a variety of Yesso scallop (*Patinopecten yessoensis*) characterized by carotenoid enrichment. Here, we found that another *CCO*-encoding gene named *PyBCO2* (β-carotene oxygenase 2) was widely expressed in *P. yessoensis* organs/tissues, with the highest expression in striated muscle. Inhibiting *BCO2* expression in *P. yessoensis* through RNA interference led to increased carotenoid (pectenolone and pectenoxanthin) deposition in the striated muscle, and the color of the striated muscle changed from white to light orange. Our results indicate that *PyBCO2* might be a candidate gene used for improving carotenoid content in normal Yesso scallops, and also in ‘Haida golden scallops’.

## 1. Introduction

Carotenoids are the most widespread color pigments found in nature and perform a host of functions in living organisms, such as being a major source of provitamin A and preventing oxidative damage [1,2,3]. As essential nutrients for humans and animals, carotenoids are primarily produced by plants, bacteria, and fungi, and are obtained by animals through their diet [4]. Therefore, a good carotenoid source can be used as a supplement to a balanced diet [5,6]. Muscle carotenoid accumulation has become an important economic trait in meat animals such as livestock, poultry, and fish, and increasing carotenoid content can improve the nutritional value and quality of meat [7,8,9]. Identifying the genes that regulate carotenoid deposition could enhance our understanding of the genetic basis of this trait, and thus the development of carotenoid-enriched animals [10].

Carotenoids are selectively absorbed, transported, and metabolized in animals, and can be enzymatically converted into a variety of products [11,12]. Carotenoid cleavage oxygenases (*CCO*s) mediate the degradation of carotenoids to colorless substances, which is a key step in carotenoid utilization [13,14]. Genetic studies in animals suggest that *CCO*s can control carotenoid homeostasis to affect carotenoid coloration in the body surface or tissues, thereby being potential candidate genes for improving carotenoid deposition [15]. In mammals, the loss of function of the *CCO* gene, named *BCO2* (β-carotene oxygenase 2), increased the content levels of carotenoids, resulting in the yellow color of milk and serum [16] as well as in fat tissues in cows [17], and a nonsense mutation (c.196C>T) of *BCO2* gene was also strongly associated with yellow fat in sheep [18]. In chickens, the *BCO2* gene affects the deposition of carotenoids in the skin and is used for the selective breeding of chickens with yellow skin to meet consumer preferences [19]. In the aquatic animal salmon, which is a famous seafood for its carotenoid-enriched muscle, *BCO2-like* controls muscle carotenoid pigmentation [20].

Carotenoid enrichment is also an important economic trait for some marine invertebrates [21,22]. In our previous study, a new variety named ‘Haida golden scallop’, which is characterized by an orange adductor muscle with carotenoid deposition, was developed for the Yesso scallop (*Patinopecten yessoensis*), representing a high-quality scallop in the market [23,24]. A *CCO* homolog, *PyBCO-like 1*, was found to be the causal gene of carotenoid enrichment in ‘Haida golden scallops’ [25]. Through transcriptome analysis, we found that another *CCO*-encoding gene, *PyBCO2*, was also highly expressed in striated muscle, suggesting that *BCO2* may be another candidate gene for improving the carotenoid content of normal Yesso scallops and also ‘Haida golden scallops’. In this study, the *PyBCO2* gene was characterized, and its possible function in carotenoid metabolism was analyzed. We inhibited *PyBCO2* expression through RNA interference (RNAi), which led to the color of striated muscle changing from white to light orange, and an increase in carotenoid (pectenolone and pectenoxanthin) deposition in striated muscle. Our results indicate the role of *BCO2* in the carotenoid metabolism of aquatic invertebrates and suggest that it could be a candidate gene for gene editing to improve muscle carotenoid content in scallops.

## 2. Results and Discussion

### 2.1. Characterization of PyBCO2 Gene Sequence

Through a transcriptome analysis of the Yesso scallop muscle, a gene named *PyBCO2* was found, which was homologous with the causal *PyBCO-like 1* gene for carotenoid coloration in ‘Haida golden scallop’ and showed high similarity with *BCO2*s in other animals. The full length of the *PyBCO2* cDNA sequence was determined to be 5583 bp, containing an open reading frame (ORF) of 1653 bp, a 5′ untranslated region (UTR) of 252 bp, and a 3’UTR of 3678 bp (Figure S1A). The ORF encoded a protein of 550 amino acids with a predicted molecular weight (MW) of about 62.2 kDa and an isoelectric point (pI) value of 6.41.

**Figure 1:**
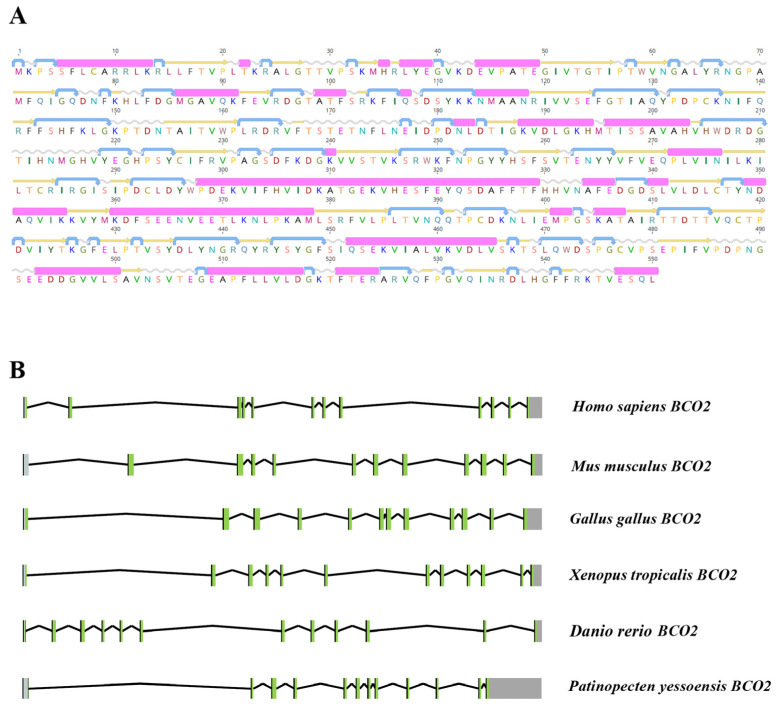
(**A**) The secondary structure of *PyBCO2* protein. The pink cylinder represents the alpha helix; the straight yellow arrow represents the beta strand; the wavy line represents the coil; the curved arrow represents the turn. (**B**) The gene structure of *BCO2*s in *P. yessoensis*, *H. sapiens*, *M. musculus*, *G. gallus*, *X. tropicalis*, and *D. rerio*. The ORF and UTR regions are shown as green boxes and grey boxes, respectively; introns are shown as fold lines.

Based on the bioinformatics analysis, *PyBCO2* protein contained the typical RPE65 domain of the *CCO* superfamily from positions 38 to 544 (Figure S1B). A secondary structure analysis revealed that *PyBCO2* protein was composed of 26 alpha helices, 40 beta strands, 45 coils, and 53 turns (Figure 1A). The subcellular localization of *PyBCO2* was analyzed by Wolf PSORT and YLoc software, both of which predicted that *PyBCO2* is located in the mitochondria. A mitochondrial import sequence (30-aa leader sequence) was found in its N-terminal extension part. This result was consistent with the subcellular localization of vertebrate BCO2, which was reported to be a mitochondrial carotenoid oxygenase with broad substrate specificity [12,26].

Furthermore, a genomic structure analysis of *PyBCO2* was conducted. The results showed that the *PyBCO2* gene contained 12 exons and 11 introns, which have the same exon/intron numbers as other vertebrate species, including *Homo sapiens*, *Mus musculus*, *Gallus gallus*, *Xenopus tropicalis*, and *Danio rerio* (Figure 1B). These results indicate that the functions of *PyBCO2* and vertebrate *BCO2* might be conserved.

### 2.2. Homology and Phylogenetic Analysis

**Figure 2:**
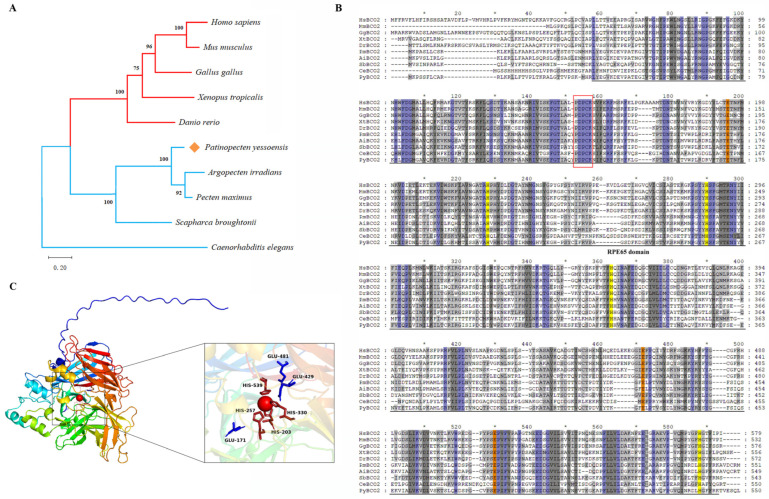
Phylogenetic analysis, sequence alignment, and protein structure of BCO2 proteins. (**A**) Phylogenetic tree of BCO2 protein in *P. yessoensis* and other species. The numbers show the bootstrap percentages (1000 replicates) obtained using the maximum-likelihood (ML) method. The vertebrate and invertebrate BCO2s are denoted by red and blue branch lines, respectively. *P. yessoensis* BCO2 is marked with an orange diamond. (**B**) Multiple alignments of amino acid sequences of BCO2 from *P. yessoensis* and other species. The shade residues in blue, dark gray, and light gray represent completely (=100%), strongly (≥75%), and weakly (≥50%) conserved residues. The conservative His residues and Glu residues are highlighted with yellow and orange backgrounds, respectively. The PDPCK motif is enclosed in a red rectangular frame. The RPE65 domain is denoted by a black line. The species abbreviations are as follows: Hs: *H. sapiens*; Mm: *M. musculus*; Gg: *G. gallus*; Xt: *X. tropicalis*; Dr: *D. rerio*; Pm: *P. maximus*; Ai: *A. irradians*; Sb: *S. broughtonii*; Ce: *C. elegans*; Py: *P. yessoensis*. (**C**) The structure and catalytic active centers of *PyBCO2* protein. The active sites, where four His residues coordinate with the Fe^2+^ and three Glu residues form the second coordination sphere, are highlighted with an enlarged black border in the right box. The Fe^2+^ ion is shown as a red sphere. The His residues and Glu residues are highlighted in red and blue, respectively.

The *PyBCO2* protein sequence was aligned with the homologous protein sequences from various species of other vertebrates and invertebrates. *PyBCO2* displayed higher identities with the BCO2 homologs of *Argopecten irradians* (73.64%) and *Pecten maximus* (78.58%), but relatively lower identities with other vertebrate species, including *H. sapiens*, *M. musculus*, *G. gallus*, *X. tropicalis*, and *D. rerio* (38.48~40.65%), and the lowest identity with *Caenorhabditis elegans* (31.62%) (Table S1), which was further supported by phylogenetic analysis (Figure 2A).

Similar to vertebrate BCO2s, *PyBCO2* exhibited all of the characteristic features of carotenoid cleavage oxygenases, including four tightly conserved His residues and three Glu residues, as well as the conserved PDPCK motif (Figure 2B) [27]. As a non-heme iron oxygenase, BCO2 cleaves the carotenoid substrate by activating molecular oxygen with a Fe^2+^ ion as a cofactor, which is coordinated by four conserved His residues, with three Glu residues forming the second coordination sphere [14]. Sequence alignment analysis confirmed the conservation of these catalytic sites in *PyBCO2*, which was further supported by tertiary structure prediction (Figure 2C), showing the structural basis of *PyBCO2* for carotenoid cleavage. The PDPCK motif in metazoan BCO2 proteins was reported to be a signature motif providing specificity for palmitoylation of the cysteine residue within this motif, and, thus, may mediate interactions with the internal membranes to capture their carotenoid substrates [28]. The conservation of catalytic sites and the PDPCK motif between *PyBCO2* and its vertebrate homologs suggests a similar function for BCO2 in scallops and higher organisms.

### 2.3. Expression Pattern of PyBCO2 in Adult Organs/Tissues

**Figure 3:**
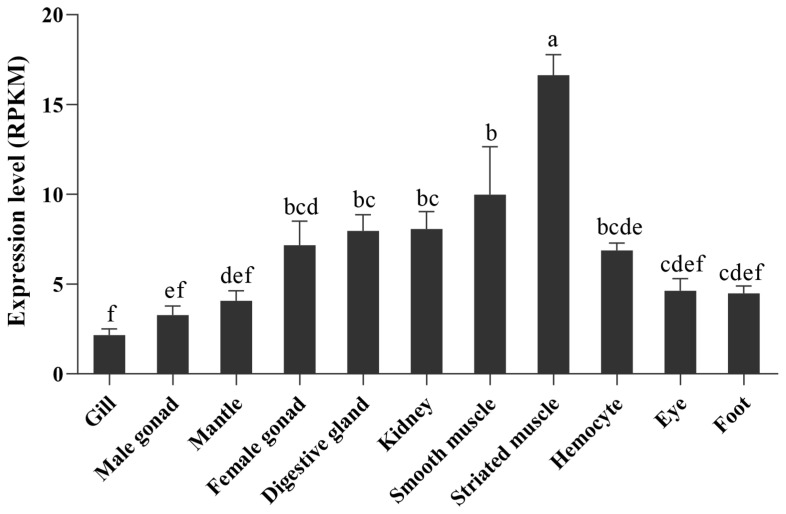
Expression of *PyBCO2* gene in adult organs/tissues of Yesso scallop. Different lowercase letters mean significant differences between organs/tissues (*p* < 0.05).

The *PyBCO2* gene was ubiquitously expressed in all examined scallop organs/tissues, with the highest expression levels detected in the striated muscle, followed by the smooth muscle (Figure 3). The average RPKM (reads per kilobase per million mapped reads) of *PyBCO2* gene expression in the female gonads, kidneys, digestive glands, and hemocytes was between 5 and 10, while that in the gills, male gonads, mantle, eyes, and feet was less than 5 (Figure 3). The widespread expression of *BCO2* was also reported in vertebrate species, such as *H. sapiens*, *G. gallus*, and *Oncorhynchus mykiss* [19,29,30]. Studies have shown that the accumulation of carotenoids can induce oxidative stress in mitochondria, and *BCO2* functions as a key regulator to prevent the adverse effects caused by excess carotenoids [31]. The main food for scallops in the sea is microalgae, such as chrysophyceae, diatoms, and chlorella, which all can produce carotenoids [32,33,34]. The wide expression of *PyBCO2* in scallop organs/tissues implies that it might be involved in the degradation of excessive carotenoids accumulated from the ingested microalgae, or in providing the metabolites of carotenoids, in the whole body. The high expression of *PyBCO2* in the striated muscle, the main tissue of scallops that is edible by humans, indicates the potential of this gene to be used in carotenoid enrichment in scallops.

### 2.4. Functional Analysis of PyBCO2

**Figure 4:**
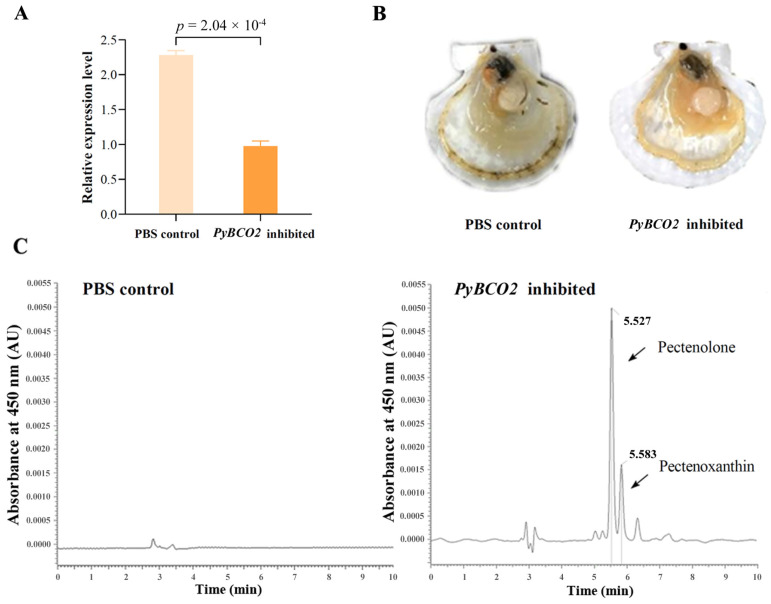
Functional verification of *PyBCO2*. (**A**) Expression of *PyBCO2* in the striated muscles of Yesso scallops in the PBS control group and the *PyBCO2* inhibited group. *p* < 0.05 indicates differences that are statistically significant. (**B**) The phenotype of the striated muscles of Yesso scallops in the PBS control group and the *PyBCO2* inhibited group. (**C**) HPLC separation of carotenoids at 450 nm in the striated muscles of Yesso scallops in the PBS control group and the *PyBCO2* inhibited group. The peaks of pectenolone and pectenoxanthin are marked by arrows with the retention time at 5.527 min and 5.583 min, respectively.

In order to verify whether *PyBCO2* has the function of degrading carotenoids, RNAi experiments were carried out by injecting *PyBCO2* dsRNA into the striated muscle of Yesso scallops. As shown in Figure 4A, the expression of *PyBCO2* in the striated muscle was significantly more reduced in the *PyBCO2* inhibited group than that in the PBS control group (*p* = 2.04 × 10^−4^), indicating that the dsRNA successfully entered into the cells in the striated muscle and *PyBCO2* expression was successfully suppressed.

The phenotypes of the Yesso scallops in the PBS control group and the *PyBCO2* inhibited group are shown in Figure 4B. The color of the striated muscles in the PBS control group was white, whereas that of the *PyBCO2* inhibited group appeared slightly orange. To determine which types of carotenoids are responsible for the orange striated muscle after *PyBCO2* inhibition, an HPLC analysis was performed. The result showed that after inhibiting *PyBCO2* expression, two obvious carotenoid peaks were detected in the striated muscle with the retention time at 5.527 min and 5.583 min, respectively, but no obvious carotenoid peaks were observed in the PBS control group (Figure 4C), suggesting that the orange color of striated muscle in the *PyBCO2* inhibited group was mainly caused by the enrichment of these two carotenoids. Further analysis based on a previously published method indicates that these two carotenoid peaks were pectenolone and pectenoxanthin, respectively [23,24]. Compared with the PBS control group without detecting any carotenoids, the peak areas of pectenolone and pectenoxanthin in the *PyBCO2* inhibited group were (1.40 × 10^4^) ± (5.40 × 10^3^) µv·s and (6.05 × 10^3^) ± (2.85 × 10^3^) µv·s, respectively, indicating that *PyBCO2* has the function of degrading pectenolone and pectenoxanthin. Additionally, no obvious phenotypic changes in the growth and development of scallops were observed compared with the control group. However, the potential impact of carotenoid accumulation on growth and development cannot be ignored. Our previous studies have found that the ‘Haida golden scallop’ with carotenoid-enriched orange muscle showed advantages in growth and stress resistance, which may be affected by carotenoid accumulation [35]. The corresponding phenotypes were not observed in this study, which may be because the time of RNAi is not enough to see these phenotypic changes in scallops.

Pectenolone and pectenoxanthin are two main carotenoids in Yesso scallops, especially in female gonads, and they are also the main carotenoids responsible for the muscle coloration of the ‘Haida golden scallop’ [23,36,37]. Pectenolone shows excellent antioxidative activity by inhibiting lipid peroxidation and contributing to protection against oxidative stress [38,39]. Pectenoxanthin (alloxanthin/cynthiaxanthin) also has beneficial effects on human health, such as anti-inflammatory activities and anti-carcinogenesis [40,41]. Increasing the content of pectenolone and pectenoxanthin in the striated muscle could largely improve the nutritional value of scallops. Our findings prove that it is a feasible approach to increasing the content of pectenolone and pectenoxanthin in scallops by decreasing the expression of *PyBCO2*. 

As a carotene-cleaving enzyme, BCO2 could catalyze the oxidative cleavage of colored carotenoids acquired from the diet into colorless apocarotenoids, which is an essential step in carotenoid degradation [12]. Numerous studies have demonstrated that missense mutations in *BCO2* or *BCO2* knockout resulted in increased carotenoid accumulation. For example, a cis-acting mutation that lowered *BCO2* expression caused domestic chickens to have yellow skin due to carotenoid accumulation [42,43]. The genetic disruption of *BCO2* function in mice resulted in increased plasma and blood accumulation of the dietary carotenoid [44]. A nonsynonymous mutation in the *BCO2* gene also induced higher carotenoid accumulation in canary tissues [45]. These studies emphasize the important role of *BCO2* in regulating carotenoid metabolism and accumulation. In this study, through inhibiting *PyBCO2* expression, the deposition of the carotenoids pectenolone and pectenoxanthin was observed in scallop muscle, indicating the function of *PyBCO2* in the bioavailability of marine carotenoids. Our result not only proves that *PyBCO2* could participate in the degradation of pectenolone and pectenoxanthin, but also provides a reliable target gene for improving carotenoid content in scallops and even other aquatic animals.

## 3. Materials and Methods

### 3.1. Biological Materials

The healthy one-year-old Yesso scallops used in this experiment were collected from natural populations by Zoneco Group Co., Ltd. (Liaoning, China), and then, transported to the laboratory. After collection, the Yesso scallops were acclimated in filtered and aerated seawater at 9–12 °C for one week and were fed *Isochrysis galbana* twice daily before the experiments.

### 3.2. RNA Isolation and cDNA Synthesis

Total RNA was extracted from the striated muscle of Yesso scallops using guanidinium isothiocyanate, as described by Hu et al. [46], and then, was digested with DNase I (TaKaRa, Shiga, Japan). RNA concentration and purity were measured by a Nanovue Plus spectrophotometer (GE Healthcare, Piscataway, NJ, USA), and the RNA integrity was checked by running electrophoresis on 1% agarose gel. RNA samples with clear bands corresponding to 18S and 28S rRNA on the gel and OD260/OD280 ratios between 1.8 and 2.0 were used for cDNA synthesis. First-strand cDNA was synthesized from 2 µg of total RNA using oligo(dT)_18_ and MMLV reverse transcriptase (TaKaRa, Shiga, Japan) in a total volume of 20 µL. The reaction was performed at 42 °C for 90 min and terminated by heating at 70 °C for 10 min. The cDNA products were diluted to 20 ng/mL and stored at −20 °C.

### 3.3. Cloning of PyBCO2 Gene and Sequence Analysis

The DNA and mRNA gene sequences of *PyBCO2* were obtained from the whole genome [47] and transcriptome sequence databases [25] of the Yesso scallop. The coding sequence of *PyBCO2* was predicted by ORF Finder (https://www.ncbi.nlm.nih.gov/orffinder/, accessed on 1 January 2018) and was confirmed by a polymerase chain reaction (PCR) with a gene-specific primer (*PyBCO2*-Fw/Rv, Table 1); the primer pairs were designed using the software Primer Premier v.5.0 [48]. Conserved domains of *PyBCO2* were analyzed using the Simple Modular Architecture Research Tool (SMART) (http://smart.embl-heidelberg.de/, accessed on 1 January 2018), and the physicochemical characteristics, including MW and pI, were predicted using ProtParam (http://br.expasy.org/, accessed on 1 January 2018). Secondary and tertiary structures were predicted using Geneious Prime (http://www.geneious.com/, accessed on 5 March 2018) and Alphafold2 (http://github.com/deepmind/alphafold, accessed on 5 March 2018), respectively. The subcellular localization of *PyBCO2* was predicted using WOLF PSORT (https://www.genscript.com/wolf-psort.html, accessed on 20 May 2020), YLoc (https://abi-services.cs.uni-tuebingen.de/yloc/webloc.cgi, accessed on 20 May 2018), iPSORT Prediction (https://ipsort.hgc.jp/predict.cgi, accessed on 20 May 2018), and Predotar (https://urgi.versailles.inra.fr/predotar/, accessed on 20 May 2018).

The *BCO2*s of various species, including *H. sapiens*, *M. musculus*, *G. gallus*, *X. tropicalis*, and *D. rerio*, were selected for comparing their gene structure with *PyBCO2*. The positional information of exons and introns was characterized by comparing the cDNA sequence to the genomic sequence. The gene structure was drawn using the R package “ggbio” [49].

Multiple sequence alignment and phylogenetic analysis were performed using *PyBCO2* and its homologous proteins from various species of other vertebrates and invertebrates, including *H. sapiens* (Q9BYV7.5), *M. musculus* (NP_573480.1), *G. gallus* (XP_040546304.1), *X. tropicalis* (AAH75500.1), *D. rerio* (NP_001035402.1), *P. maximus* (XP_033763808.1), *A. irradians*, *S. broughtonii*, and *C. elegans* (NP_494694.2). The BCO2 protein sequences were aligned using the ClustalX program [50], and alignment editing was performed using the GeneDoc multiple sequence alignment editor (http://www.nrbsc.org/gfx/genedoc/index.html, accessed on 20 May 2018). The amino acid sequence identity between *PyBCO2* and its homologous protein of other species was calculated using BLAST (http://blast.ncbi.nlm.nih.gov/Blast.cgi, accessed on 20 May 2018). A phylogenetic tree was constructed using the maximum-likelihood (ML) method with the LG model using MEGA X software [51]. Bootstrapping with 1000 replications was performed to evaluate the phylogenetic tree.

### 3.4. Expression Analysis of PyBCO2 in Different Adult Organs/Tissues

*PyBCO2* gene expression in adult organs/tissues, including the mantle, gill, male gonad, female gonad, kidney, digestive gland, smooth muscle, striated muscle, foot, eye, and hemocyte, was analyzed using transcriptomic data from Yesso scallops [47]. The expression of *PyBCO2* was measured as RPKM values.

### 3.5. Inhibiting PyBCO2 Expression

To verify the function of *PyBCO2* in carotenoid degradation, a dsRNA-mediated RNAi experiment was performed. The sequence of the *PyBCO2* gene was submitted to siDirect 2.0 (http://sidirect2.rnai.jp/, accessed on 12 September 2018) for prediction and screening of the RNAi target sequence. siDirect 2.0 is an online server that utilizes a fast and sensitive homology search algorithm to minimize any off-target effects and ensure functional dsRNA design. In addition, the RNAi target sequence that was uniquely aligned with the Yesso scallop genome was selected for further dsRNA synthesis. *PyBCO2* dsRNA was produced using the MEGAscript^®^ RNAi Kit (Thermo, Waltham, MA, USA) following the manufacturer’s protocol. The acclimated Yesso scallops were randomly divided into two groups (PBS control group and *PyBCO2* inhibited group). For each individual, 100 μL of specific dsRNA (1 μg/μL, for *PyBCO2* inhibited group) or l× PBS (for PBS control group) was injected into the striated muscle. Each scallop was injected once a week for four weeks. The striated muscles of the two scallop groups (PBS control group and *PyBCO2* inhibited group) were sampled for gene expression analysis and carotenoid extraction.

### 3.6. Quantitative Real-Time PCR (qRT-PCR)

The expression pattern of *PyBCO2* after inhibiting *PyBCO2* expression was analyzed by qRT-PCR. The cDNAs of striated muscles of Yesso scallops from the *PyBCO2* inhibited group and the PBS control group were used as templates. Gene-specific primers (*PyBCO2*-qPCR-Fw/Rv, Table 1) were designed using Primer Premier v5.0 [48].

qRT-PCR was performed on a Light-Cycler Roche 480 Real-time PCR System (Roche Applied Science, Mannheim, Germany). The qRT-PCR reactions were carried out in a total volume of 20 μL containing 2× Light Cycler 480 SYBR Green I Master (Roche Applied Science, Mannheim, Germany), 0.4 μL of each primer (10 μM), 2 μL of diluted cDNA template (10 ng/μL), and 7.2 μL of nuclease-free water. The running program was as follows: 94 °C for 10 min, followed by 40 cycles of 94 °C for 15 s and 62 °C for 1 min. Melting curve analysis was performed to verify that the primer set amplified a single product. Ubiquitin (*UBQ*) was used as an internal control to normalize gene expression. The relative expression level of *PyBCO2* was calculated using the 2^−ΔΔCt^ method [52].

### 3.7. High-Performance Liquid Chromatography (HPLC) Analysis

The carotenoids of striated muscles in scallops after *PyBCO2* inhibition were analyzed using reversed-phase HPLC, as previously described [23,24], with minor modifications. The solvents and chemicals used for carotenoid extraction were on the scale of analytical reagents. To avoid the degradation and isomerization of carotenoids, amber glassware was used and all the extraction procedures were carried out in an aphotic environment. Briefly, 1 g of striated muscle was lyophilized and extracted three times with 4 mL of methanol containing 0.1% butylated hydroxytoluene (BHT). After drying the methanol with nitrogen, the residue was dissolved in 2 mL of a mobile phase solution (acetonitrile/methanol/dichloromethane, 50:46:4, *v*/*v*/*v*) and filtered through a 0.22 μm nylon-6 membrane (13 mm, Solarbio, Beijing, China). HPLC analysis of carotenoids was performed using a Chromaster (HITACHI, Tokyo, Japan) equipped with an analytical-scale LaChrom C18 column (4.6 × 250 mm, 5 μm inner diameter; HITACHI, Tokyo, Japan). The mobile phase for chromatographic separation was a ternary, isocratic solvent system of acetonitrile/methanol/dichloromethane at a ratio of 50:46:4. The other detail parameters were as follows: a 10 μL injection volume; a 25 °C column temperature; a 1.0 mL per minute flow rate; a 450 nm UV-Vis detector; and a 10 min separation time. Peak area (μv·s) was used as a relative quantification for inter-group comparison. 

### 3.8. Statistical Analysis

All statistical analyses were performed using SPSS 19.0 software [53]. Significant differences in *PyBCO2* expression among adult organs/tissues were analyzed by one-way analysis of variance. The expression difference of *PyBCO2* in the striated muscles between the PBS control group and *PyBCO2* inhibited group were determined using a dependent-samples t-test. *p* values less than 0.05 were considered statistically significant.

## 4. Conclusions

A gene encoding carotenoid cleavage oxygenase, *PyBCO2*, was identified in Yesso scallops. We provided evidence that *PyBCO2* is widely expressed in scallop organs/tissues, with the highest expression in the striated muscle, the main edible tissue of scallops. Functional studies showed that the inhibition of *PyBCO2* expression led to the deposition of the scallop carotenoids pectenolone and pectenoxanthin in the striated muscle. Our results demonstrated that *PyBCO2* plays a key role in carotenoid metabolism in Yesso scallops, and it could be used in the breeding of carotenoid-enriched scallops, including further improving carotenoid content in ‘Haida golden scallop’.

## Figures and Tables

**Table 1:** Primers used in this study.

Primer Name	Sequence	Sequence Information
*PyBCO2*-Fw	ATGAAGCCATCATCATTTCT	Sequence validation
*PyBCO2*-Rv	CTATAACTGACTCTCTACAGT	Sequence validation
RNAi-Fw	GATCACTAATACGACTCACTATAGGGGCGACGGAACCATCCACAA	RNAi
RNAi-Rv	GATCACTAATACGACTCACTATAGGGCCTCACTGAAATCCTTCATATACACCT	RNAi
*PyBCO2*-qPCR-Fw	GATGCCAGGCTCTAAAGCAAC	qRT-PCR
*PyBCO2*-qPCR-Rv	CTGAACCCGTACGAGTAACGATACT	qRT-PCR
UBQ-Fw	TCGCTGTAGTCTCCAGGATTGC	qRT-PCR
UBQ-Rv	TCGCCACATACCCTCCCAC	qRT-PCR

## Data Availability

The data are contained within the article.

## Supplementary Materials

The following supporting information can be downloaded at: https://www.mdpi.com/article/10.3390/ijms25073947/s1.
