# Supplementary material for: Functional Analysis of β-Carotene Oxygenase 2 (BCO2) Gene in Yesso Scallop (Patinopecten yessoensis)
Source: Int J Mol Sci. 2024 Apr 2;25(7):3947. doi: 10.3390/ijms25073947 (PMC11012205; doi:10.3390/ijms25073947)
Supplement: Supplementary file 1 [file ijms-25-03947-s001.zip › Table S1.pdf]

Table S1. Comparison of the identities of BCO2 homologs between *P. yessoensis* and other organisms.

**Species**	**Full Protein (%)**
Patinopecten yessoensis	100
Homo sapiens	39.49
Mus musculus	39.73
Gallus gallus	38.48
Xenopus tropicalis	39.58
Danio rerio	40.65
Argopecten irradians	73.64
Pecten maximus	78.58
Scapharca broughtonii	50.65
Caenorhabditis elegans	31.62
