# Supplementary figures and images for: Functional Analysis of β-Carotene Oxygenase 2 (BCO2) Gene in Yesso Scallop (Patinopecten yessoensis)
Source: Int J Mol Sci. 2024 Apr 2;25(7):3947. doi: 10.3390/ijms25073947 (PMC11012205; doi:10.3390/ijms25073947)

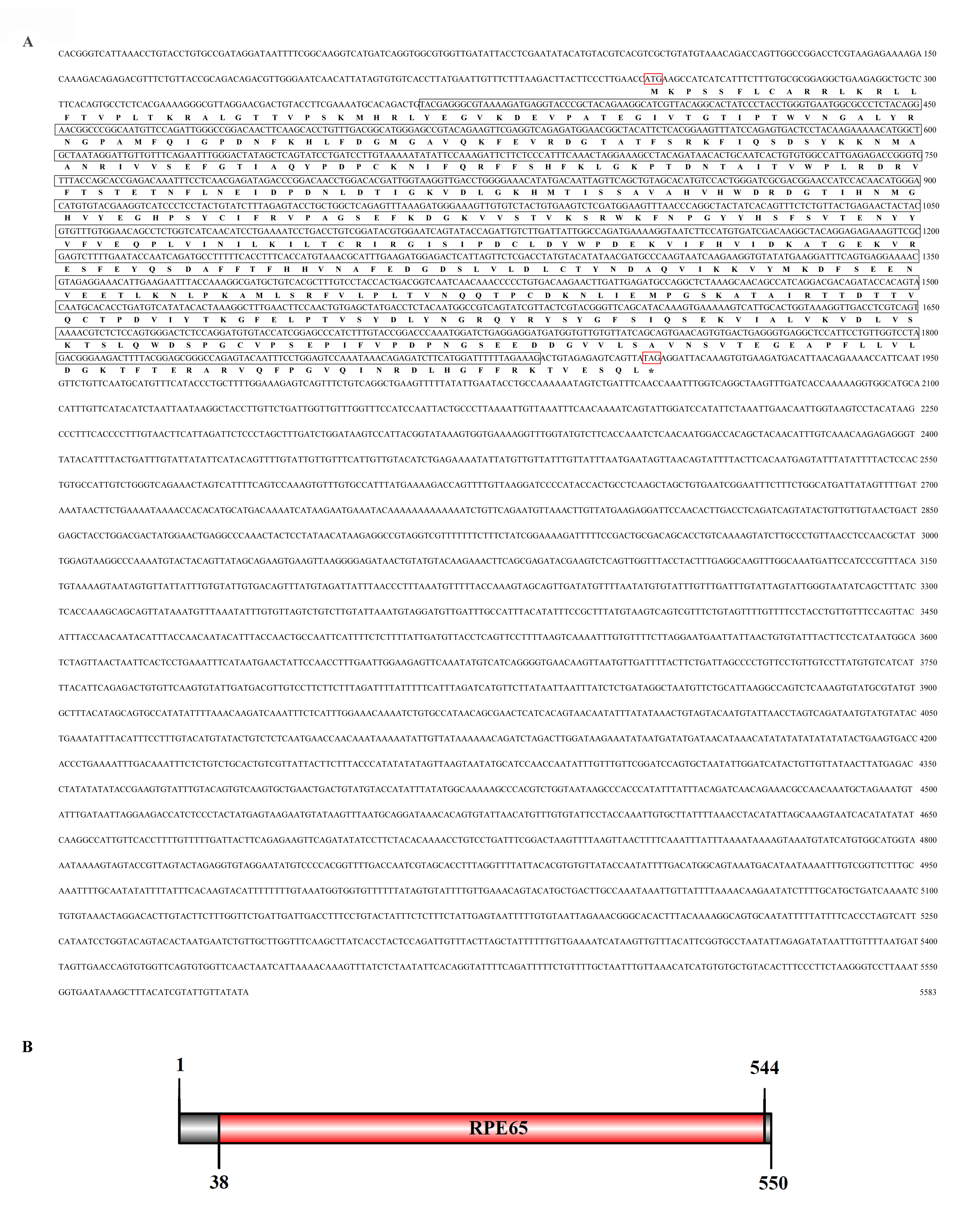

Supplement: Supplementary file 1 [file ijms-25-03947-s001.zip › Figure S1.tif]
